# Lung microbiome alterations correlate with immune imbalance in non-small cell lung cancer

**DOI:** 10.3389/fimmu.2025.1589843

**Published:** 2025-05-14

**Authors:** Jiuling Cheng, Huaqi Wang

**Affiliations:** Department of Respiratory Medicine, The First Affiliated Hospital of Zhengzhou University, Zhengzhou, Henan, China

**Keywords:** non-small cell lung cancer, lung microbiota, *Prevotella*, *Veillonella*, systemic immune-inflammation index, CD8 + T cell

## Abstract

**Background:**

Current understanding of the link between microbiota imbalance and immune function in non-small cell lung cancer (NSCLC) has not been fully elucidated. This study aims to explore the link between dysbiotic lung microbiota and immunity in NSCLC, which may provide valuable information for disease progression monitoring and prognosis prediction.

**Methods:**

Lung microbial communities from both the tumor-affected (n = 43) and contralateral healthy sides (n = 38) of lung cancer patients were analyzed by 16S rRNA sequencing. The association between microbial abundance and tumor stages, metastasis or not, nodule size, PD-L1 expression, as well as Ki-67 levels was conducted. Mann-Whitney tests were used to evaluate differences in the systemic immune-inflammation index (SII), T cell subsets (CD3^+^, CD4^+^, CD8^+^), as well as the CD4^+^/CD8^+^ ratio between different microbial expression patterns of *Prevotella* and *Veillonella*.

**Results:**

Significant β-diversity differences were observed between the tumor-bearing and contralateral normal lungs in individuals diagnosed with lung carcinoma. A notable increase in *Prevotella* (*P* = 0.044) and *Veillonella* (*P* = 0.02) was detected within NSCLC-affected lungs, whereas *Pseudomonas* (*P* = 0.008) as well as *Staphylococcus* (*P* = 0.033) were significantly reduced. Increased levels of *Veillonella* were detected in NSCLC patients at stage IIIB-IV and were positively correlated with Ki-67 expression. Furthermore, patients with higher abundance of *Prevotella* and *Veillonella* exhibited a significantly elevated systemic immune-inflammation index (SII) compared to the lower-abundance group (*P* = 0.0329), while their CD8^+^ T cell levels were significantly decreased in the higher abundance group (*P* = 0.0027).

**Conclusion:**

Microbial composition differed significantly between the tumor-affected and healthy sides in lung cancer patients. *Veillonella* was more abundant NSCLC patients at stage IIIB-IV, while increased *Prevotella* and *Veillonella* abundance correlated positively with SII but negatively with CD8^+^ T cell levels. These findings provide valuable insights into tumor-associated microbiota for monitoring disease advancement, treatment stratification and prognostic assessment.

## 1 Introduction

As the deadliest malignancy worldwide, lung cancer has a poor prognosis, with a 5-year survival probability of just 27% (1–3). Given its high mortality, lung cancer warrants the refinement of monitoring and therapeutic strategies. Beyond genetic and environmental factors, the lung microbiome has gained attention for its influence on lung cancer progression and immune modulation. It has been shown that dysbiotic commensal microbial communities may exist in the respiratory tract of lung cancer (4–7). For example, several studies have reported that lung cancer cases are associated with an increased presence of *Capnocytophaga*, *Selenomonas*, and *Veillonella* in both saliva and sputum (8–10). The dysbiotic microbial landscape associated with lung cancer remains inadequately explored.

Numerous studies established immune cell dysregulation is closely linked to the prognosis of lung cancer (11, 12). Peripheral blood immune cells have been utilized for biomarkers in disease monitoring and prognosis evaluation, offering a feasible, prompt, and non-invasive method (13, 14). The Systemic Immune-Inflammation Index (SII) is recognized as a potential parameter in disease prognosis, determined by the formula: SII = platelet count × neutrophil count/lymphocyte count (15). SII can reflect systemic inflammation and immune status, which is associated with worse overall survival (OS) and progression-free survival (PFS) of lung cancer patients (16, 17). CD8^+^ T cells serve as the primary cytotoxic effectors in antitumor immunity, and their depletion or functional exhaustion is closely linked to tumor progression and immune evasion (18–20).

Using 16S rRNA sequencing, this study investigated lung microbiota dysbiosis in lung cancer by comparing bronchoalveolar lavage fluid (BALF) from tumor-affected and contralateral healthy lungs. Additionally, we analyzed the association between dysbiotic lung microbiota and key immune and disease progression markers, including SII, CD8^+^ T cell levels, Ki-67, and tumor stage. This research sought to investigate the connection of lung microbiota imbalance with immune dysregulation in lung cancer, providing insights for cancer progression monitoring, treatment stratification and prognosis prediction.

## 2 Materials and methods

### 2.1 Study participant recruitment

Between November 2021 and June 2022, we collected 102 BALF samples at Zhengzhou University’s First Affiliated Hospital. After quality control, bronchoalveolar lavage fluid samples of both the tumor-affected (n = 43) and contralateral healthy sides (n = 38) from lung cancer patients were selected for 16S rRNA sequencing analysis. Eight samples were excluded due to 16S rRNA amplification failure. All enrolled participants were newly diagnosed with lung cancer, had no history of cancer treatment, and had not used antibiotics in the last four weeks. Additionally, individuals with a prior diagnosis of cancer were removed from this study (21). We collected clinical data, such as pathological diagnosis and tumor stage. Additionally, we assessed nodule features (size and location) and laboratory parameters, such as blood routine tests, inflammatory markers, tumor markers, and absolute counts of T-cell subsets. Immunohistochemical markers and gene mutation profiles were analyzed in paraffin-embedded lung tissue specimens. The research received ethical approval from the Ethics Committee of Scientific Research and Clinical Trials of the First Affiliated Hospital of Zhengzhou University (No. 2024-KY-0348-003), and all enrolled patients signed an informed consent form.

### 2.2 Sample collection

BALF samples were collected by an experienced clinician following standard fiberoptic bronchoscopy protocols (22, 23), with strict precautions to minimize oral contamination. Samples were obtained from both the tumor-affected and corresponding healthy lung lobes, with 10–15 mL collected from each side per patient (24). The tumor-affected side was designated as the lung cancer group, while the contralateral healthy side was defined as the control group.

### 2.3 Microbial 16S rRNA analysis

DNA was isolated from BALF and subsequently subjected to 16S rRNA sequencing. For 16S rRNA sequencing, the hypervariable V3-V4 regions were subjected to PCR using primers 341F and 806R (CCTAYGGGRBGCASCAG, GGACTACNNGGGTATCTAAT). Following PCR product purification, the library quality was evaluated prior to sequencing (25).

Paired-end reads were processed into raw tags with FLASH (Version 1.2.11) (26) to generate raw tags. Quality filtering was performed under specific conditions using the fastp (v0.23.1) quality control procedure to acquire high-quality filtered tags (27). Using the UCHIME algorithm, clean tags were aligned to the Silva database to filter out chimeric sequences, yielding the final effective tags.

### 2.4 Sequencing analysis

Taxonomic annotation was performed using QIIME2 with the Silva 138.1 reference database. Community richness, diversity, and evenness were measured by QIIME2. β-diversity was quantified by calculating both weighted and unweighted UniFrac distances. Significant differences in species composition across taxonomic levels were identified utilizing MetaStat and t-tests in R (v4.0.3). LEfSe analysis was employed to identify biomarkers, with an LDA score threshold set to 4 in the LEfSe software.

### 2.5 Data analysis and statistical methods

Statistical tests were performed using SPSS (IBM SPSS 26.0, SPSS Inc.), applying the Mann-Whitney U test or t-test for two-group comparisons of continuous variables, while categorical variables were assessed using the χ² test. Results were expressed as mean ± SD. Abundance, α-diversity, and β-diversity analyses between groups were assessed via the Mann-Whitney test. Heat maps and relationship heat maps were generated using R Version 4.3.2 software. Correlation analysis was conducted using Spearman correlation methods. A threshold of *P*-value less than 0.05 was defined as statistically significant.

## 3 Results

### 3.1 Patient features

**Figure 1:**
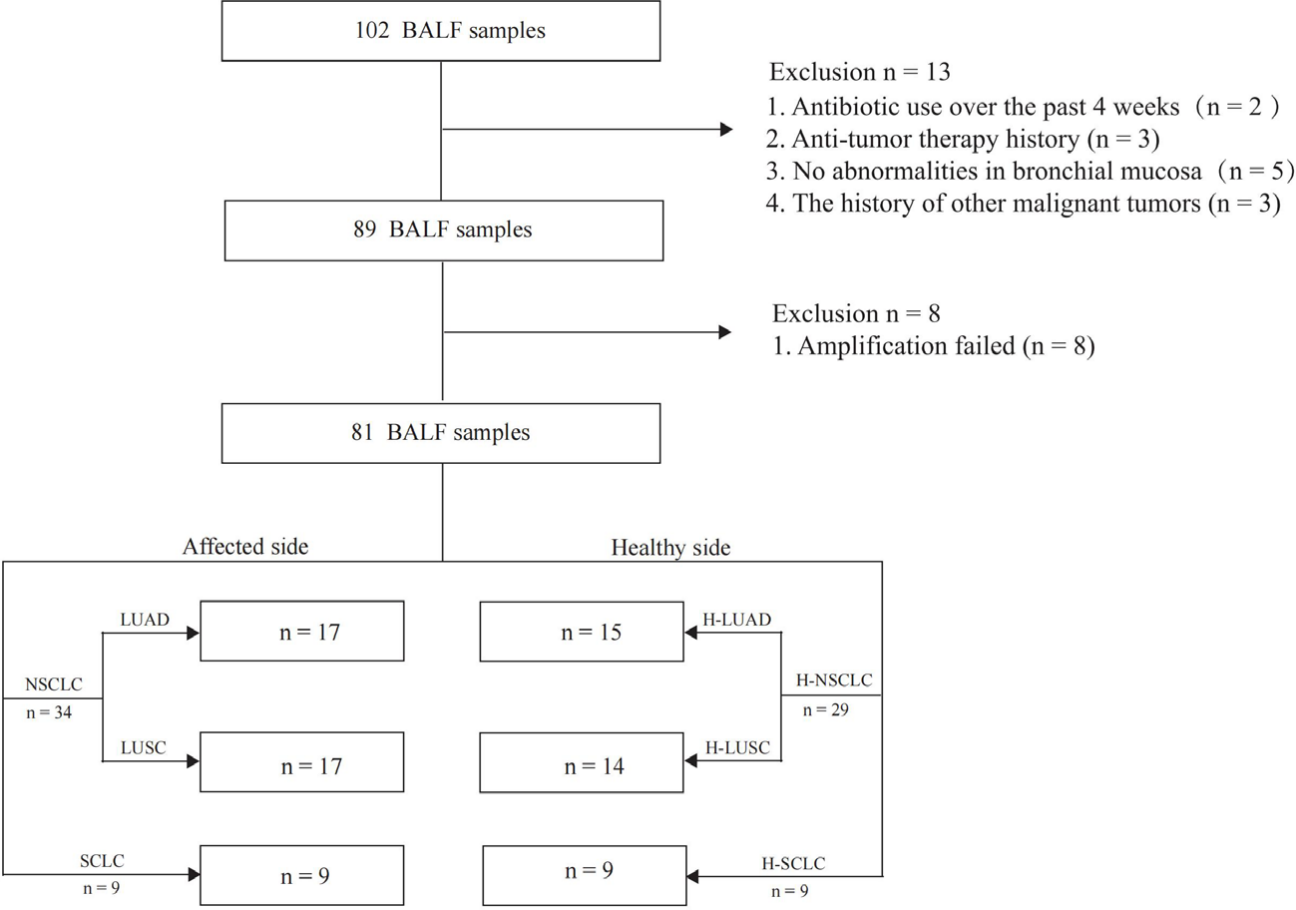
Graphical representation of study design.

81 BALF samples were included in the final analysis, with the study flowchart depicted in **Figure 1**. The median age of the lung cancer group was 62 (38–79) years, while it was 64 (44–79) years in the control group. The enrolled patients included 17 with LUAD, 17 with LUSC, and 9 with SCLC. Among the enrolled patients, 7 were at stage I, 3 at stage II, 10 at stage III, and 14 at stage IV. Three patients were diagnosed with limited-stage and six with extensive-stage SCLC. Distant metastases were detected in 31 patients, whereas 12 had non-metastatic disease. Genomic mutation data were retrieved from 23 patients (**Table 1**).

**Table 1:** Clinical characteristics of study participants.

Characteristic	Lung cancer (n = 43)	Control (n = 38)	*P* value
Sex, n (%)			
Female	10 (23.2)	11 (29.0)	1.00
Male	33 (76.8)	27 (71.0)	1.00
Median age (range), years	62 (38 - 79)	64 (44 - 79)	1.00
BMI (kg/m^2^) (mean ± SD)	23.5 ± 2.5	23.3 ± 2.8	1.00
Smoking history, n (%)			
Non-smokers	15 (35)	15 (39)	1.00
Smokers	28 (65)	23 (61)	1.00
Smoking pack-years (mean ± SD)	24.7 ± 26.1	23.3 ± 25.7	1.00
Histology type, n (%)			
LUAD	17 (39.5)	15 (39.5)	1.00
LUSC	17 (39.5)	14 (36.8)	1.00
SCLC	9 (21.0)	9 (23.7)	1.00
Tumor stage, n (%)			
I	7 (16.3)	7 (18.4)	1.00
II	3 (6.9)	4 (10.5)	1.00
III	10 (23.3)	8 (21)	1.00
IV	14 (32.6)	10 (26.3)	1.00
Limited stage	3 (6.9)	3 (8.0)	1.00
Extensive stage	6 (14.0)	6 (15.8)	1.00
Tumor metastasis, n (%)			
Metastasis	31 (72)	25 (65.8)	1.00
Non-metastasis	12 (28)	13 (34.2)	1.00
Lesion location, n (%)			
Upper left	11 (25.5)	10 (26.3)	1.00
Lower left	15 (34.9)	12 (31.5)	1.00
Upper right	6 (14.0)	6 (15.8)	1.00
Middle right	3 (7.0)	2 (5.3)	1.00
Lower right	8 (18.6)	8 (21.1)	1.00
Blood cell count(mean ± SD)			
Total white blood cells (×109/L)	7.40 ± 2.13	7.36 ± 2.19	1.00
Neutrophils (×109/L)	4.86 ± 1.54	4.85 ± 1.62	1.00
Eosinophils (×109/L)	0.19 ± 0.27	0.20 ± 0.28	1.00
Basophils (×109/L)	0.03 ± 0.02	0.03 ± 0.01	1.00
Monocytes (×109/L)	0.57 ± 0.30	0.56 ± 0.31	1.00
Lymphocytes (×109/L)	1.62 ± 0.90	1.67 ± 0.93	1.00
Gene mutation, n (%)			
EGFR	7 (46.7)	6 (50.0)	1.00
ALK	1 (6.7)	0 (0)	1.00
KRAS	3 (20.0)	2 (16.7)	1.00
PTEN	1 (6.7)	1 (8.3)	1.00
TP53	4 (26.7)	2 (16.7)	1.00
MAP2K1	2 (13.3)	2 (16.7)	1.00
CDKN2A	1 (6.7)	0 (0)	1.00
PIK3CA	1 (6.7)	0 (0)	1.00
ERBB2	1 (6.7)	0 (0)	1.00
AKT1	1 (6.7)	0 (0)	1.00
HER	1 (6.7)	1 (8.3)	1.00

### 3.2 Microbial composition of BALF in lung cancer

**Figure 2:**
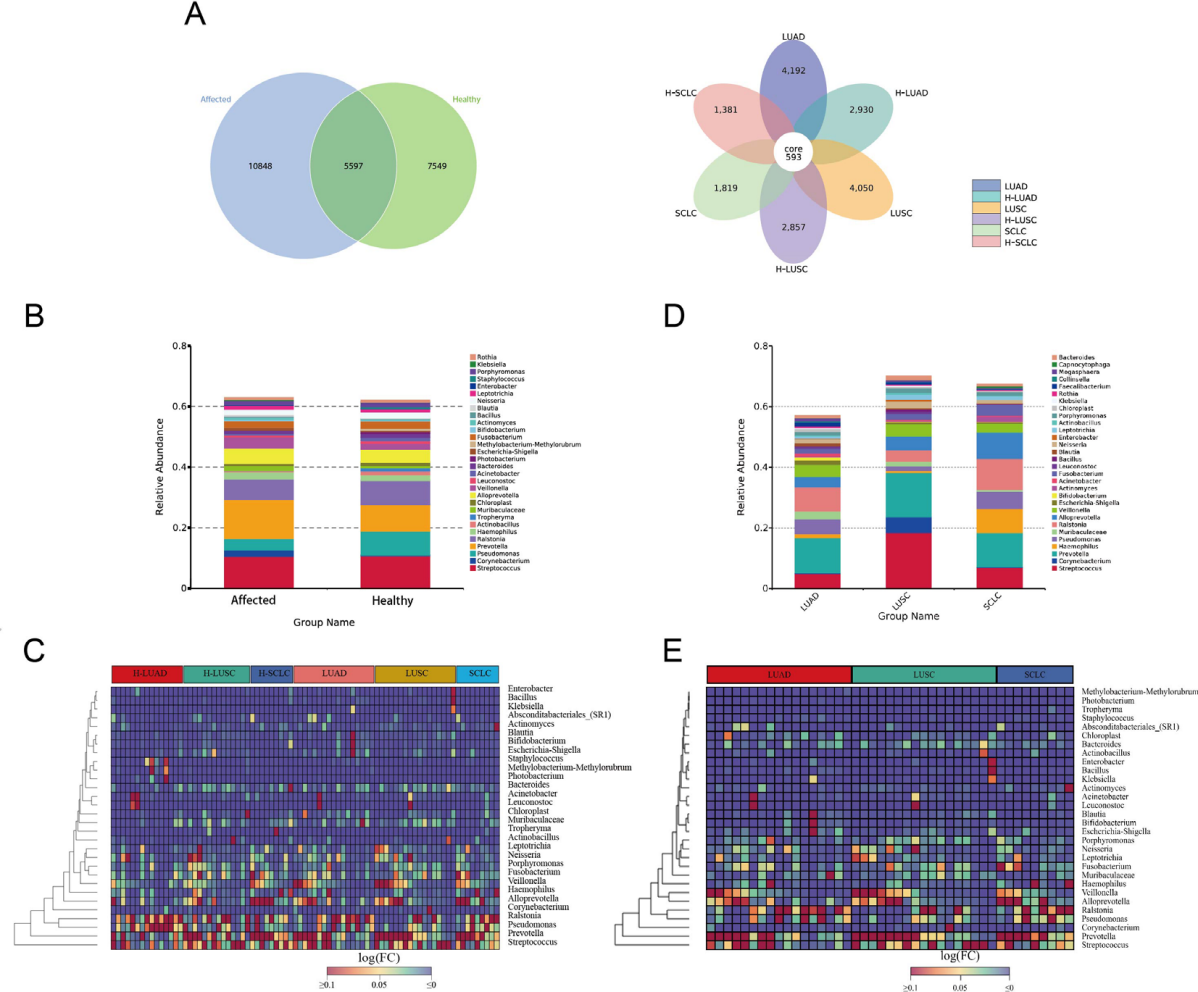
Microbial composition of BALF in lung cancer. **(A)** Venn diagrams illustrate the shared and unique ASVs (Amplicon Sequence Variants) between the tumor-affected and healthy sides across different pathological subtypes of lung cancer. **(B, C)** Genus-Level Taxonomic Profile of the BALF Microbiome, comparing the affected and healthy sides of lung cancer. **(D, E)** Taxonomic profiles of BALF microbiomes across different pathological subtypes of lung cancer. The affected side includes NSCLC, LUAD, LUSC, and SCLC. The corresponding healthy sides are denoted as H-NSCLC, H-LUAD, H-LUSC, and H-SCLC, respectively.

A total of 23,994 ASVs were identified, comprising 16,445 ASVs from the affected lung segment and 13,146 ASVs from the contralateral healthy lung segment (**Figure 2A**). Microbial communities in the lower airway differ between the tumor-affected and contralateral healthy sides in lung cancer patients. The top 10 genera, *Prevotella*, *Streptococcus*, *Ralstonia*, *Alloprevotella*, *Veillonella*, *Pseudomonas*, *Neisseria*, and *Fusobacterium*, were prevalent on both affected and contralateral healthy sides. *Prevotella*, *Veillonella*, *Corynebacterium*, and *Muribaculaceae* were notably abundant in cancer-affected individuals, while *Pseudomonas*, *Porphyromonas*, and *Actinobacillus* were predominant in the contralateral healthy group (**Figures 2B, C**, **Supplementary Figure S1**).

The microbial composition differs significantly across various pathological subtypes of lung cancer. In individuals diagnosed with LUAD and LUSC, the ten taxa with the highest prevalence are *Prevotella*, *Ralstonia*, *Streptococcus*, *Pseudomonas*, *Veillonella*, *Alloprevotella*, *Muribaculaceae*, *Fusobacterium*, *Escherichia-Shigella*, and *Neisseria*. In contrast, the top 10 genera in small cell lung cancer (SCLC) are *Prevotella*, *Ralstonia*, *Alloprevotella*, *Haemophilus*, *Streptococcus*, *Pseudomonas*, *Fusobacterium*, *Veillonella*, *Actinomyces*, and *Leptotrichia* (**Figures 2D, E**).

### 3.3 Biodiversity analysis demonstrates divergent microbial community compositions between the tumor-affected and contralateral healthy sides

**Figure 3:**
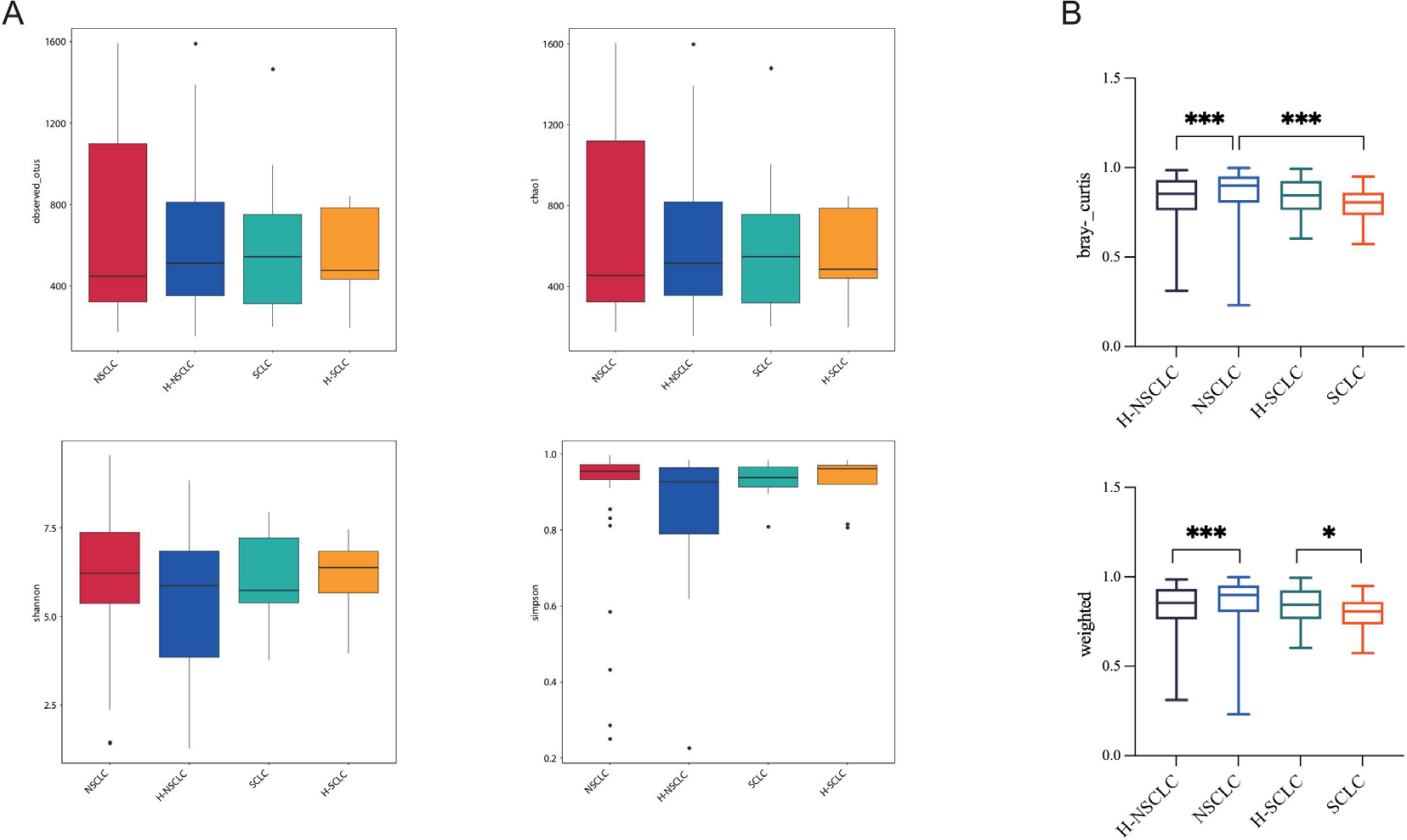
Biodiversity analysis demonstrates divergent microbial community compositions between the tumor-affected and contralateral healthy sides. **(A)** Observed OTUs, Chao1, Shannon, and Simpson indices were used to analyze microbial α-diversity. **(B)** β-diversity comparison of BALF microbiota between the tumor-affected and healthy sides in NSCLC and SCLC, as measured by weighted UniFrac distances and Bray-Curtis dissimilarity indices. *P-value < 0.05; ***P-value < 0.001.

The tumor-affected and contralateral healthy sides exhibited no significant differences in α-diversity, as evaluated by Chao1, Observed OTUs, Shannon, and Simpson indices (**Figure 3A**). However, distinct β-diversity patterns were observed between tumor-affected and contralateral healthy sides in NSCLC, supported by weighted UniFrac and Bray-Curtis indices (**Figure 3B**). These observations highlight the microbial composition and community structure of the tumor-affected lung that differ significantly from those of the contralateral healthy lung segment.

### 3.4 Differentially abundant microbes in lung cancer dysbiosis

**Figure 4:**
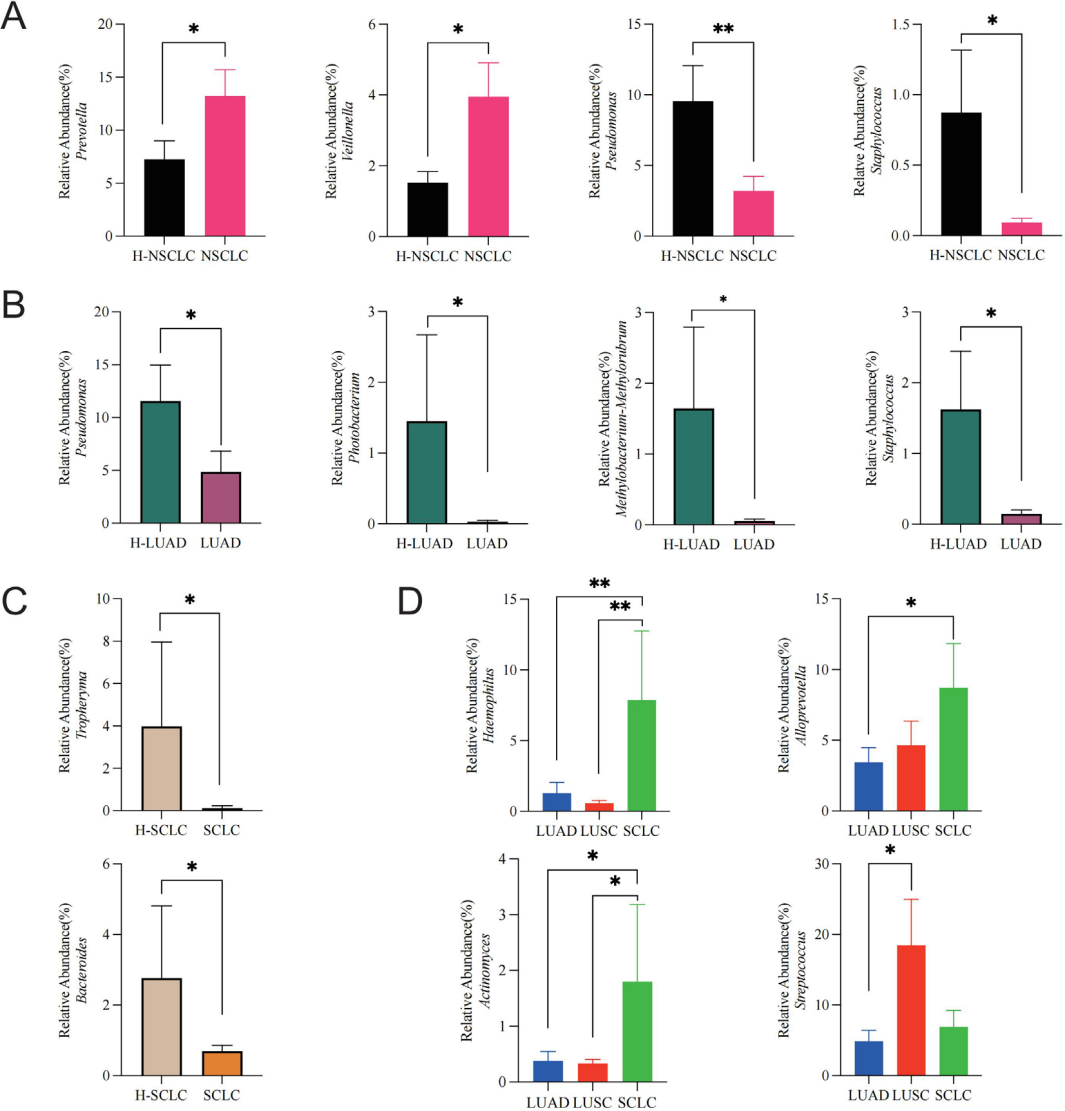
Lung cancer microbiome dysbiosis profiles. **(A)** Comparison between the tumor-affected and healthy sides in NSCLC, **(B)** LUAD, **(C)** SCLC, and **(D)** across different pathological subtypes of lung cancer. *P-value < 0.05; **P-value < 0.01.

We investigated microbial differences between the tumor-affected and contralateral healthy sides in NSCLC to characterize lung cancer-associated dysbiosis. Compared to the contralateral healthy side, the NSCLC-affected side displayed an enrichment of *Prevotella* (*P* = 0.044) and *Veillonella* (*P* = 0.02), whereas *Pseudomonas* (*P* = 0.008) as well as *Staphylococcus* (*P* = 0.033) displayed a notable decrease (**Figure 4A**).

To assess subtype-specific dysbiosis, we further analyzed microbial differences between the tumor-affected and contralateral healthy sides across distinct pathological subtypes. In LUAD, *Pseudomonas* (*P* = 0.042), *Photobacterium* (*P* = 0.049), *Methylobacterium-Methylorubrum* (*P* = 0.02), and *Staphylococcus* (*P* = 0.003) were markedly more abundant on the healthy side (**Figure 4B**). Similarly, in SCLC, *Tropheryma* (*P* = 0.035) and *Bacteroides* (*P* = 0.048) exhibited higher abundances on the healthy side (**Figure 4C**).

Furthermore, to explore inter-subtype differences in dysbiosis, we compared microbial profiles among tumor-affected lung segments of different pathological subtypes. *Haemophilus* was significantly enriched in SCLC compared to LUAD (*P* = 0.008) and LUSC (*P* = 0.003). *Alloprevotella* abundance was higher in SCLC compared to LUAD (*P* = 0.041). *Actinomyces* was markedly enriched in SCLC relative to LUAD (*P* = 0.017) and LUSC (*P* = 0.014). Additionally, *Streptococcus* was significantly enriched in LUSC relative to LUAD (*P* = 0.022) (**Figure 4D**).

### 3.5 Association between *Veillonella* abundance and tumor stage, proliferation, and PD-L1

**Figure 5:**
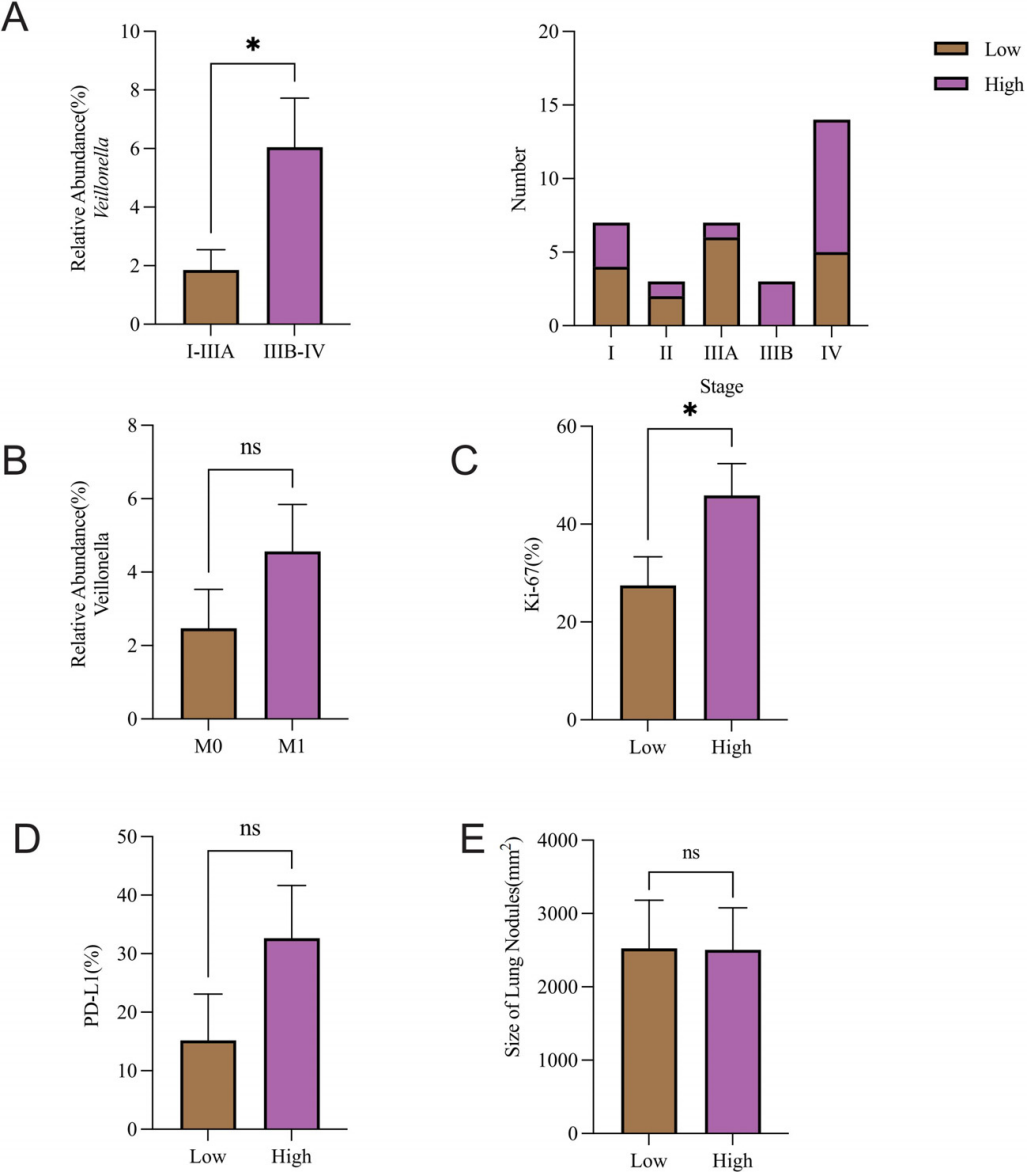
Association between *Veillonella* abundance and tumor stage, proliferation, and PD-L1. Association of *Veillonella* relative abundance with **(A)** pathological stage and **(B)** metastasis. Comparative analysis of **(C)** Ki-67 expression, **(D)** PD-L1 expression, and **(E)** Lung nodule size across *Veillonella* expression groups was evaluated. *P-value < 0.05, ns P-value > 0.05.

To explore the clinical relevance of microbial alterations, we analyzed their associations with key tumor-related indices and *Veillonella* abundance. The relative abundance of *Veillonella* was markedly elevated in NSCLC patients (stage IIIB-IV) relative to patients (stage I-IIIA) (*P* = 0.0266). Notably, the *Veillonella*-low group had a higher fraction of early-stage patients, whereas the *Veillonella*-high group was predominantly composed of late-stage patients (**Figure 5A**). Furthermore, when the relative abundance of *Veillonella* was categorized into high and low groups, the high group demonstrated a significantly greater Ki-67 expression level compared to the low group (**Figure 5C**). Additionally, a trend of higher PD-L1 expression was detected in *Veillonella*-rich subjects (**Figure 5D**), and *Veillonella* abundance appeared greater in NSCLC patients with distant metastases compared to the M0 group. However, neither difference reached statistical significance (**Figure 5B**). Lung nodule size and *Veillonella* abundance were not notably related (**Figure 5E**).

### 3.6 Altered commensal microbiota is linked to immune system disturbances and systemic inflammation in NSCLC

**Figure 6:**
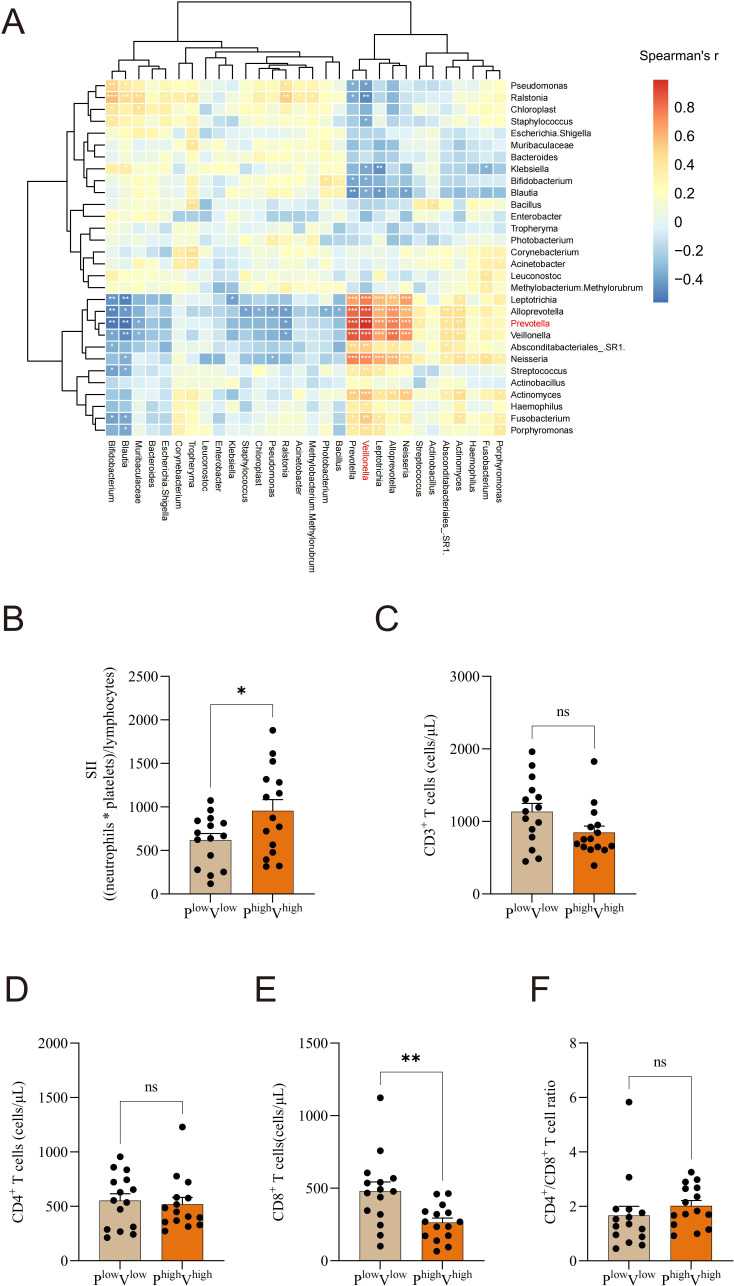
Altered commensal microbiota is linked to immune system disturbances and systemic inflammation in NSCLC. **(A)** Spearman correlation analysis of the top 30 microbial taxa, **(B)** Based on *Prevotella* and *Veillonella* median levels, individuals were classified into high- and low-abundance groups. The systemic immune-inflammation index (SII) was compared between the high-abundance *Prevotella* and *Veillonella* group and the low-abundance group. **(C)** CD3^+^ T cells, **(D)** CD4^+^ T cells, **(E)** CD8^+^ T cells, and **(F)** CD4^+^/CD8^+^ T cell ratio levels were compared between the high-abundance *Prevotella* and *Veillonella* group and the low-abundance group. *P-value < 0.05; **P-value < 0.01, ***P-value < 0.001, ns P-value > 0.05.

To investigate the relationship between microbial abundance and immune-inflammatory responses, we analyzed the immune profiles of patients stratified by *Prevotella* and *Veillonella* abundance. Spearman correlation analysis of the top 30 microbial taxa revealed that *Prevotella* exhibited a substantial positive association with *Veillonella* (**Figure 6A**). According to the median abundance of *Prevotella* and *Veillonella*, patients were grouped into high and low abundance categories; SII was markedly elevated in the high-abundance group relative to the low-abundance group (**Figure 6B**). Furthermore, CD8^+^ T cell levels were significantly lower in the high-abundance *Prevotella* and *Veillonella* group compared to the low-abundance group (**Figure 6E**). Analysis revealed no significant differences between the two groups in terms of CD3^+^ T cells, CD4^+^ T cells, or the CD4^+^/CD8^+^ T cell ratio (**Figure 6F**).

## 4 Discussion

Lung microbial dysbiosis has been implicated in lung cancer (28), yet its relationship with tumor progression and immunity remains incompletely understood. Investigating this interplay is essential for elucidating the potential role of microbial alterations in cancer development. In this study, we collected BALF from both the tumor-affected and contralateral healthy lungs of newly diagnosed lung cancer patients and performed 16S rRNA sequencing to profile microbial communities. We further analyzed microbial diversity, identified dysbiotic microbial taxa, and explored their associations with tumor stage, metastasis, and systemic immune status. Our findings contribute to a more profound understanding of lung cancer-associated microbial dysbiosis and its potential relevance to disease progression and immune modulation.

In this study, instead of using healthy individuals as controls, we selected the contralateral healthy lung of the same patient as an internal control, thereby minimizing inter-individual heterogeneity (29–31). Consistent with the findings from other studies (29, 32), our results confirm that no notable variable in α-diversity was found between the tumor-affected and contralateral healthy sides from lung cancer patients. However, distinct microbial β-diversity patterns between the tumor-affected and contralateral healthy sides underscore the importance of analyzing both sites to understand disease-associated microbial alterations.

Previous studies have demonstrated that bacterial composition and abundance differ among different pathological types (33–35). In our study, we observed that the top 10 most abundant bacterial genera were largely similar between LUAD and LUSC, whereas SCLC exhibited distinct microbial characteristics. The results emphasize the clinical significance of microbiome-based stratification in lung cancer subtypes.

Accumulating evidence highlights microbial dysbiosis is closely linked to cancer onset, progression, and patient outcomes (36–38). In our study, we observed an enrichment of *Prevotella* and *Veillonella* from the tumor-affected BALF of lung cancer patients. Similarly, Wen Zeng et al. reported a marked rise in *Prevotella* and *Veillonella* from lung cancer patients, further supporting our findings (39). Previous studies have demonstrated that *Prevotella* facilitates tumorigenesis in diverse malignancies, including lung cancer through upregulation of pro-inflammatory cytokines (e.g., IL-1β) and oral squamous cell carcinoma via suppression of tumor suppressor gene expression and tumor microenvironment remodeling (21, 40). Additionally, it accelerates breast cancer progression through the depletion of the host’s intrinsic indole-3-pyruvic acid (41). Notably, experimental evidence also supports these mechanisms, as *Prevotella copri*-colonized mice develop marked inflammation and immune dysregulation (42), reinforcing the link between *Prevotella*, immunity and cancer progression. While *Veillonella* has been recognized as a promising marker for disease assessment and classification (8, 43). Furthermore, *Veillonella* has been associated with activation of tumor-promoting pathways, including PI3K (21, 44–47) and the Nod2/CCN4/NF-κB axis, which may contribute to inflammatory responses and proliferation in non-small cell lung adenocarcinoma. *V. parvula* mediates activation of the Nod2/CCN4/NF-κB signaling pathway to promote non-small cell lung adenocarcinoma progression (48). Additionally, *V. parvula* has been linked to induce B cells in the tumor microenvironment, potentially promoting colorectal tumor development (49). While our study cannot fully resolve the causal direction between microbial dysbiosis and immune dysregulation, experimental models (e.g., *Prevotella*-colonized mice developing inflammation) and their tumor-promoting mechanisms (e.g., NF-κB-mediated cytokine upregulation) suggest that dysbiosis may contribute to immune dysregulation in lung cancer. Further longitudinal studies are warranted to validate this hypothesis.

In contrast, we noted a substantial decline in *Pseudomonas* and *Staphylococcus* abundance in the tumor-affected lung compared to the contralateral healthy side. Interestingly, recent studies have highlighted the potential anticancer properties of *Pseudomonas aeruginosa*, particularly through azurin, a protein known to inhibit tumor growth (50). Genetically modified *P. aeruginosa* strains have been shown to induce cancer cell death, inhibit proliferative signaling pathways, and activate anti-tumor immune responses (51–58). This study raises the intriguing possibility that the depletion of *Pseudomonas* in lung cancer patients may alter the tumor microenvironment in a way that facilitates cancer progression. However, more research is essential to illuminate the underlying biological pathways. While the reduced Staphylococcus abundance challenges its previously reported pro-tumorigenic role (59, 60), it highlights the complexity of host-microbiome interactions in lung cancer progression.

Microbiota significantly contribute to tumor progression. Our study report that *Veillonella* abundance is significantly higher in lung cancer patients at stage IIIB-IV (typically not surgery-eligible), compared with those at stage I-IIIA (usually surgery-eligible). This discovery offers a new potential biological marker for helping assess surgical eligibility in NSCLC patients, enabling more precise guidance for clinical treatment strategies. Similarly, previous research has demonstrated significant differences in fecal microbiota between early- and late-stage melanoma patients (61). In our study, NSCLC patients with distant metastases (M1) exhibited higher *Veillonella* abundance than the M0 group, though the difference wasn’t statistically significant, probably owing to the limited sample size. This warrants verification through large-cohort studies. Furthermore, our study identified that patients with higher *Veillonella* abundance exhibited a significant increase in Ki-67 expression, a well-established marker of cellular proliferation (62, 63). *Veillonella* is known to be involved in lactate metabolism (64), and excessive lactate accumulation has been shown to trigger the activation of the HIF-1α, leading to enhanced tumor proliferation (65, 66). These findings suggest that *Veillonella* may potentially contribute to a tumor-promoting microenvironment by modulating lactate metabolism and hypoxia-related signaling pathways. From a translational medicine viewpoint, detecting specific microbial taxa in BALF, like *Prevotella* and *Veillonella*, could allow for helping develop treatment plans. Moreover, these microbial biomarkers have prognostic potential. For example, the correlation between *Veillonella* abundance and tumor stage/Ki-67 expression indicates it could monitor disease progression and predict prognoses.

Systemic inflammation is a hallmark of cancer progression (67), and an elevated systemic immune-inflammation index (SII) is associated with poor prognosis in NSCLC (68–72). In our study, *Prevotella* and *Veillonella* abundance was positively correlated with SII, suggesting a link to a pro-inflammatory state. CD8^+^ T cells are key to anti-tumor immunity (73). The observed negative correlation between *Prevotella* and *Veillonella* abundance and CD8^+^ T cell levels suggests potential immune suppression. Further research is needed to determine whether *Prevotella* and *Veillonella* actively promote tumor progression or serve as biomarkers of disease severity. The link between *Prevotella*, *Veillonella*, systemic inflammation, and immune suppression suggests that these microbial features might predict immune therapy responses, helping clinicians guide patient stratification and develop personalized treatment regimens.

This study has several limitations. First, while 16S rRNA sequencing provided taxonomic profiling of BALF microbiota, its resolution is insufficient to identify microbial species or strains, which may obscure functionally distinct subgroups. Future studies should employ shotgun metagenomic sequencing (mNGS) or culture-based strain isolation to resolve microbial genomic heterogeneity and validate their immunomodulatory roles. Second, the small cohort of SCLC patients limits the statistical power to detect clinically meaningful associations. Multi-center studies with expanded sample sizes are warranted to confirm the generalizability of our findings. Third, although significant correlations were observed between dysbiosis (e.g., *Prevotella*/*Veillonella* enrichment) and immune dysregulation, these associations do not establish causality. Mechanistic validation using germ-free murine models colonized with patient-derived microbiota or *in vitro* co-culture systems is critical to delineate microbial-immune crosstalk in the future. Lastly, while microbiota-targeted interventions (e.g., probiotics, phage therapy, or precision antibiotics) represent promising adjuvant strategies for NSCLC, their clinical efficacy and safety require rigorous evaluation.

In conclusion, our study demonstrates that tumor-affected lower airways experience a significant disruption in microbial homeostasis, relative to the contralateral healthy lower airway in lung cancer patients. By utilizing the contralateral lung as an internal control, we provide a refined analysis of microbial alterations, revealing bacterial taxa that may be associated with tumor progression through inflammation and immune modulation. Specifically, *Prevotella* and *Veillonella* were enriched in tumor-affected lungs and correlated with systemic inflammation and immune suppression. These results provide a framework for upcoming studies on microbiome-based biomarkers for disease monitoring and prognostic assessment in lung cancer.

## Data Availability

The datasets presented in this study can be found in online repositories. The names of the repository/repositories and accession number(s) can be found below: https://www.ncbi.nlm.nih.gov/bioproject/?term=PRJNA1134686.
