# Supplementary material for: Lung microbiome alterations correlate with immune imbalance in non-small cell lung cancer
Source: Front Immunol. 2025 May 14;16:1589843. doi: 10.3389/fimmu.2025.1589843 (PMC12086715; doi:10.3389/fimmu.2025.1589843)
Supplement: Supplementary file 1 [file DataSheet1.docx]

Supplementary Material

# Supplementary Figures

**
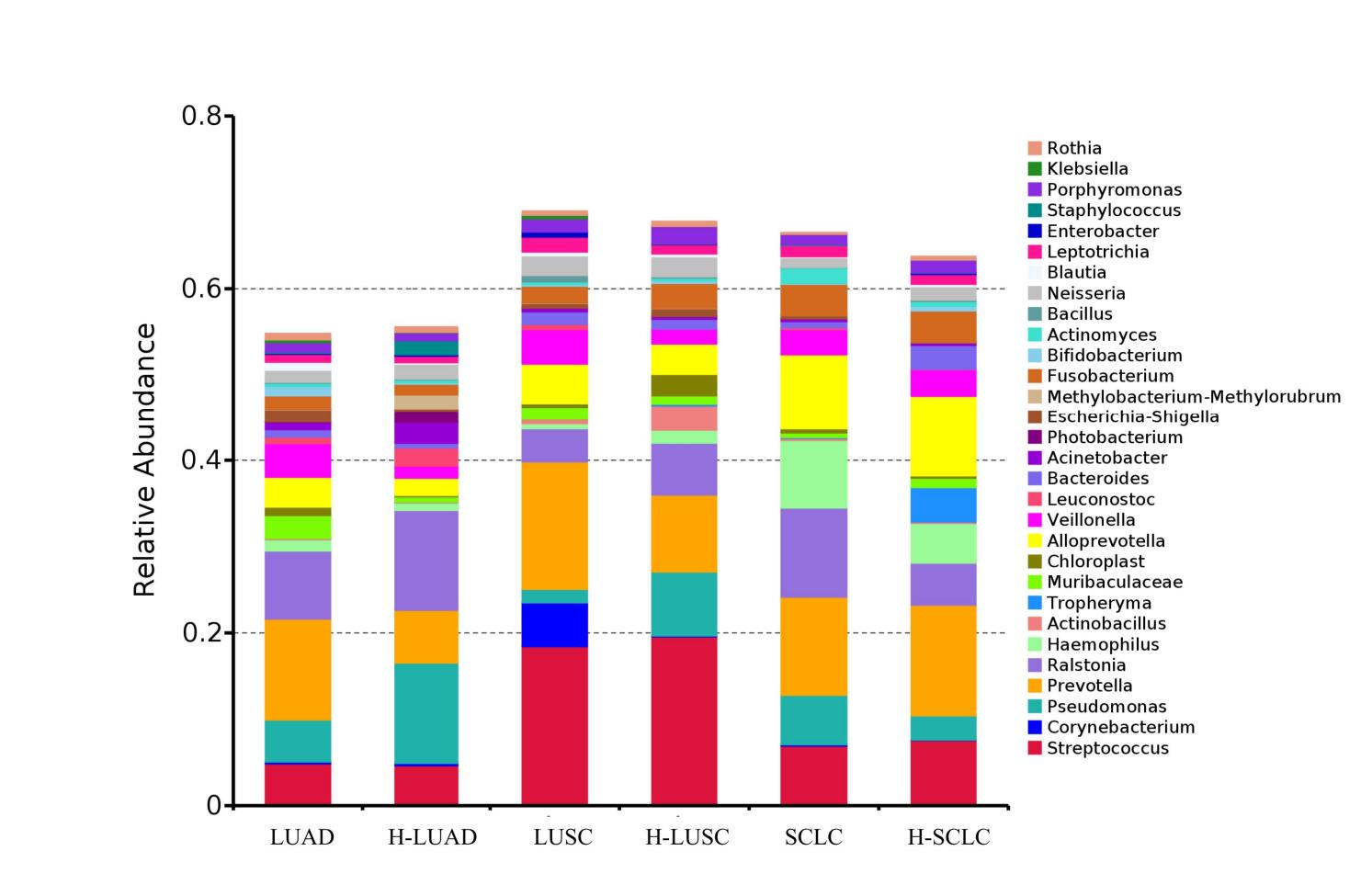
**

**Supplementary Figure 1.** Genus with the Highest Relative Abundance Across Different Pathological Types.
